# Findings from a qualitative analysis: Social media influencers of color as trusted messengers of HPV vaccination messages

**DOI:** 10.1371/journal.pone.0319160

**Published:** 2025-04-04

**Authors:** Amelia Burke-Garcia, Erin Cutroneo, Dasha Afanaseva, Kayla Madden, Angela Sustaita-Ruiz, Preethi Selvan, Estefany Rivera Sanchez, Amy Leader

**Affiliations:** 1 Public Health Research Department, NORC at the University of Chicago, Washington, DC, United States of America; 2 Division of Supportive Medicine, Medical Oncology, Thomas Jefferson University, Philadelphia, Pennsylvania, United States of America; 3 Brilla Media, North Bay Village, Florida, United States of America; 4 Division of Population Science, Medical Oncology, Thomas Jefferson University, Philadelphia, Pennsylvania, United States of America; University of Wyoming, UNITED STATES OF AMERICA

## Abstract

**Background:**

Despite HPV vaccination reducing the prevalence of cervical cancer by 90%, vaccination rates remain lower among communities of color due to vaccine hesitancy and mistrust in traditional public health messengers. The emergence of social media influencers, a newer kind of messenger, presents a unique opportunity to share immunization messages in new ways with a variety of communities. This paper reviews the qualitative findings from a study aimed at assessing influencers’ perceptions of and approaches to sharing messaging about the HPV vaccine.

**Methods:**

Guided by several theories (Theory of Planned Behavior, Narrative Theory, and Opinion Leader Theory), the study team designed an intervention-based study consisting of qualitative interviews and social media influencer-designed and disseminated messaging. We worked closely with an influencer marketing firm to recruit 10 influencers of color who had children aged 9-14 – to write about the vaccine with their followers. Influencers used a provided factsheet to draft social media posts about vaccinating their children against HPV. Influencers were interviewed about their post and posts and interview transcripts were analyzed for key themes.

**Results:**

Most influencers were hesitant to talk about vaccinations for fear of backlash. Most committed to writing, however, because they were compelled to support important health topics. All used the power of storytelling to convey the messages and highlighted their personal journeys of vaccine decision making. Influencers also highlighted the struggles of parenting and talked emotionally about how making decisions about this vaccine prompted feelings about their child growing up. Influencers also believed that they could help people make the decision to vaccinate.

**Conclusions:**

The findings from this study elucidates the emotional context within which parents are being asked to vaccinate their children and thus, how personal the decision to vaccinate is. Most influencers noted that they had received a doctor’s recommendation but were taking the time to do their own research. Insights from this study can help inform current and future public health communication programs aimed at supporting immunization efforts. It also can provide lessons for other health topics.

## Introduction

In 2019, the World Health Organization identified vaccine resistance and hesitancy as a top threat to global health [1]. Vaccine hesitancy, defined as patient-level reluctance to receive vaccines, can be conceived on a spectrum that spans from cautious acceptors to outright deniers [2]. Hesitancy is often fueled by concerns about vaccine safety and efficacy, which can be undermined by widespread circulating disinformation and misinformation [3]. Furthermore, vaccine hesitancy is rooted in medical mistrust, which is higher among historically marginalized communities due to generations of systemic and institutional racism, health inequities, and misrepresentation in research [4].

Growth in social media platforms and channels has dramatically changed the nature of communication. There are currently 4.95 billion social media users globally [5], and upwards of 90% of Americans use social media to connect with one another, engage with news content and share information [6]. Today, there is near-equal participation on social media by age, race, gender, education, and income levels [7], which ensures a wide range and diversity of experiences, opinions, and discourse. An aspect of social media that has emerged in recent years are “online influencers,” which are “everyday people who are incredibly influential within their online social networks” [8]. They often garner large followings on social media, which increases the likelihood of engagement with their posts, which, in turn, amplifies the visibility of their content [9]. For years, online influencers have been recruited to disseminate information and encourage consumer behaviors [10–12], as they are viewed as highly credible and trustworthy messengers [13]. Only recently have health communication and behavioral scientists begun to shift their attention to partnering with online influencers to spread credible health messages [14–16].

The rise of social media has democratized the ability for many voices, including lay consumers, to share their knowledge about and experiences with vaccination, in both positively and negatively framed messages. To date, a variety of studies have shown that anti-vaccine messages receive more attention across social media platforms than pro-vaccine messages [17–21]. Semantic network analysis has allowed for the graphical representation of these discussions, showing that pro-vaccine posts and anti-vaccine posts feature different key words and cluster together, albeit independently [22]. Lastly, research shows that social media may impact vaccine knowledge, awareness, and attitudes among people who read this information and may be influential in vaccine uptake [23]. As the field of social media research continues to grow, greater insight into the impact of these messages and messengers on behavior must be better understood.

The historic public health crisis resulting from the SARS-CoV-2 (COVID-19) pandemic not only resulted in significant morbidity and mortality in the U.S. and around the world, but it also caused disruptions to many essential health services, including the provision of routine immunizations [24]. Moreover, these disruptions disproportionately impacted racial and ethnic minority communities [25,26].

One of these essential and highly effective immunizations under threat is the HPV vaccine. According to a recent study, the prevalence of HPV infection in the US was 40% overall, and the prevalence of HPV-related disease was 24.2% in males and 19.9% in females [27]. Long-term HPV infection increases the risk of several kinds of cancer affecting both men and women, including throat cancer, penile cancer, cervical cancer and more [28,29]). The HPV vaccination has been shown to prevent cervical cancer by 97% [30], and recent studies have found a 12% decline in cervical cancer among US women younger than 25 between 2012 to 2019, and a pursuant steep decline in cervical cancer mortality among this population between 2016 and 2021 [31]. The COVID-19 pandemic’s disruption to routine HPV vaccination could lead to a reversal of this trend and, and without an aggressive effort to get vaccination back on track, a rise in cervical and other cancers.

Additionally, studies of HPV vaccine uptake in adults have revealed that adult Hispanics have lower odds of receiving the vaccine than non-Hispanic Whites and Non-Hispanic Blacks [32,33]. As well, data suggest that Asians have low rates of HPV vaccine initiation and completion, and that Native Americans have both a higher burden of HPV-associated cancers than other racial and ethnic groups as well as low uptake of the HPV vaccine [34,35]. Despite Non-Hispanic Blacks having a higher rate of vaccination, both Non-Hispanic Blacks and Hispanic populations have higher incidences of cervical cancer than Non-Hispanic White populations, and Black women had the highest overall mortality rates [32,33]. For this reason, it is essential to approach promotion of the vaccine through a lens of equity and inclusion. Moreover, as the vaccine is most effective when administered before the age of 16 [36], it is essential to reach parents of children of these ages in order to make this important decision on their behalf. This moment in time provides a unique opportunity to learn from social media influencers, as trusted messengers for communities of color, to better understand the messages they create about the HPV vaccine, and what resonates with their online communities to overcome vaccine hesitancy.

It is within this context that Thomas Jefferson University (TJU) and NORC at the University of Chicago (NORC) (referred to as “the study team” throughout this paper) collaborated to design this study.

### Aims

This study sought to understand social media influencers perceptions about HPV vaccination and how they craft messages about the HPV vaccine for their followers. It also sought to understand how influential they think they are with their followers. This paper describes the methods and results of this intervention-based study, as well as implications for theory and practice.

### Evaluation questions

This study identified three key evaluation questions (EQ):

EQ1: How did the influencers write about getting their child vaccinated against HPV in their posts?
EQ2: How clearly did the influencers recommend the HPV vaccine in their posts?
EQ3: How did the influencers perceive themselves in terms of their ability to be persuasive about vaccination to their followers?

## Methods

This study employed an intervention-based design. Following best practices set forth in community-based participatory research, the study team partnered with 10 social media influencers who almost exclusively write for Black, Hispanic, or Native American audiences to co-create one post encouraging vaccination based on an HPV vaccination fact sheet provided to them, but written in their own words.

After the post was created, but before the post went live, the study team interviewed each influencer to understand how they created their post to speak to their audience. The number of interviews was selected based on best practices for qualitative interviewing documented in the literature [37–39]. Saturation was achieved through discussion and agreement amongst the study team. Posts were also content analyzed for word choice, message framing, and image and video content (where applicable).

The study team conducted this study in 2022, collecting qualitative data from these two main sources: 1) interviews with social media influencers, and 2) thematic analysis of influencers’ social media posts. Recruitment took place between May 18, 2022 and October 19, 2022. Both TJU and NORC’s Institutional Review Boards reviewed and approved the study’s procedures and protocols (#152,153,2405 and #22-03-701, respectively).

### Study administration and implementation

#### Instrument and material development.

The study team created a package of materials for the study. | First, in collaboration with Brilla Media, they created recruitment materials. This included the Influencer Recruitment Email (S1 Appendix) as a template for outreach to influencers in Brilla Media’s network, as well as the Recruitment/Screening Questions for Influencer Recruitment (S2 Appendix), which was sent to interested influencer to determine their eligibility for the project. The team then developed materials for the influencer interviews, including the Verbal Consent Statement for Telephone Interview (S3 Appendix) and Influencer Interview Guide (S4 Appendix). Finally, the study team developed resources to guide influencers in crafting their posts and recruiting their followers to a pre- post- survey about the post. This included the HPV Vaccine Research Study: Vaccine Hesitancy Project Guidelines (S5 Appendix), as well as Facts at your Fingertips: HPV information for influencers to reference in post (S6 Appendix), the latter of which was tailored by cancer experts at TJU to include factual and relevant information that influencers could adapt to use in their posts.

#### Study participant screening criteria.

The study team identified the following criteria for participant inclusion in the study:

1. Hispanic, Black, Asian, or Native American parents or caregivers of children between the ages of 9-14.
2. Influencers that regularly developed quality content that would align with the overall project theme (i.e., health and wellness, vaccination awareness, parenting, etc.).
3. Influencers were also screened based on their own acceptance of childhood vaccines, in which those who replied that they were “very hesitant” during the screening process were considered ineligible.

Given the nature of our population of study, e.g., social media influencers, the team identified additional criteria for inclusion to ensure that the influencers were actively participating online by posting content regularly and engaging with their online communities. These were:

1. Influencers had to have at least a 1% engagement rate.
2. They had to have posted once within the last 3 months (although the final selected influencers posted at least 2-3 times per week)

#### Study participant recruitment.

For this study, 10 influencers were recruited to be participants in this study. The study team worked with Brilla Media, a multicultural influencer marketing agency, to identify these. To do this, Brilla Media initially sent an alert to their network of over 100 multicultural influencers about the opportunity to participate in a new social media program (Appendix A); this contained general information about the project. In addition to this initial call, Brilla Media also reached out to influencers with whom they had existing relationships, worked before on similar campaigns, or found by searching their network and reviewing their online profiles for relevancy to the project criteria.

#### Participant screening and confirmation.

Brilla Media then compiled a list of all influencers that met study criteria for review. This included links to social platforms, followers/reach, and any other notes or insights from Brilla Media. The study team then reviewed and approved the influencers who would formally be invited to participate. Upon approval, Brilla Media sent a follow-up email with additional details (Appendix E) about the project, including deliverables, timing, and compensation to confirm influencer participation in the project. Influencers that were interested signed up using a screening form (Appendix B) to express interest, and their responses were received directly by the study team to review for eligibility.

#### Influencer post creation.

Once formally engaged to participate in the study, influencers were asked to develop a social media post containing pro-vaccine messages that would reach and resonate with their communities. Influencers were encouraged to develop content in their own tone and style that overall supported vaccination of children 9-14 against HPV. To assist them in this process, the study team provided them with a fact sheet (Appendix F) on HPV and the HPV vaccine to use as a resource when creating their social media posts. Influencers submitted their posts to Brilla Media via email to be used to inform the interviews with them. Content of these posts was never edited or revised by the study team; they were only reviewed to ensure accuracy of the posts. Once all influencer interviews were completed, the influencers were permitted to publish their post.

#### Influencer interviews.

Following the creation and review of their post – but before it went live – the study team invited the influencers to participate in a 45-minute interview with study team members. The study team wanted to interview them before the posts went live to 1) ensure there was no bias during the interview process in case influencers happened to see one another’s posts after they went live, and 2) ask them a few questions about any anticipated audience reactions prior to the posts going live. In this interview, the study team explored their background using social media and being an influencer; their overall content development process (e.g., what they write about, how they choose their topics, and how they frame their messages); how influential they think they are with their followers; their thoughts and opinions on HPV and the vaccine; and their specific approach to writing the vaccine post for this study. To guide this conversation, the study team created a semi-structured interview guide that covered these topics during which a brief consent statement was read to confirm the influencers’ participation in the study. See Appendix D.

#### Interview and post transcription and translation.

Influencers’ interviews were transcribed through the Ubiqus transcription service. Two influencer posts (participants 8 and 10) were written in Spanish, so they were de-identified and translated through a partnership between TJU and a community-based agency, Nationalities Service Center.

### Analytical process

#### Codebook development.

After the interviews were complete, the team collaborated on creating two codebooks:

1. Interview Analysis Codebook
2. A Content Post Analysis Codebook

These were developed both inductively and deductively. They consisted of several a priori codes but also allowed for other codes to be generated during the open coding process [40–43]. To develop the codebooks, the team used themes and keywords as identified in the public health communication and vaccine hesitancy-related literature. This was then supplemented by adding additional new codes that the team found to be naturally emerging in the data.

The team then went through several rounds of review of the codebooks to ensure that the team aligned on the codes and scope of the codebooks, therefore ensuring that they were appropriate tools for analysis. Additionally, a sample of content (Interviews 1 & 2 and Posts 1 & 2) was analyzed by three independent coders and then compared for analysis. The study team met to review coding discrepancies, resolve these differences through consensus, and make final adjustments to the codebooks. Kappa scores ranged from 0.42 to 0.86.

#### Coding and cleaning data.

All posts and interviews were then imported into NVIVO 1.6.1 and the study team used the two final codebooks to code the remaining interview transcripts and posts, with 2 coders assigned to each document. After coding was complete, coded files were merged among reviewers and exported. One member of the team then cleaned these exported files to remove duplicate codes.

#### Thematic analysis of coded data.

After coded files had been cleaned, the team used an analysis method derived from van Manen’s “selective approach” [41], Strauss and Corbin’s constant comparative method [42], and Banning’s ecological sentence synthesis approach [43] to ensure the research can be more easily translated into practice. As well, this approach has been documented in the literature [44,45]. First, each study team member reviewed the files individually, drawing out key themes that each saw emerging in the data. They, then, met for a thematic brainstorming session to discuss each team member’s findings and areas of agreement and disagreement. Through this process, the final key themes and learnings were identified in the data (interviews and posts)and confirmed through team consensus.

## Results

### Demographic characteristics of influencers

All 10 influencers were female; 4 identified as Black/African American and 6 identified as Hispanic. 8 influencers reported being married, while two reported being single, engaged, divorced, or widowed. Eight of the 10 influencers reported having a college or graduate degree. Six influencers reported having 1-2 children, three influencers had 3-4 children, and one influencer had 5 or more children. Five of the influencers had 1 or more children between the ages of 0 and 8, five had one or more children between the ages of 9 and 12 and eight had one or more children 13 or older. Five of the influencers reported that they were “not at all hesitant” about childhood shots, while three reported they were “not too hesitant” and two reported being “somewhat hesitant.”

Six influencers reported that at least one of their children had gotten the HPV vaccine, while 3 said none of their children had gotten the vaccine. One said they were unsure if any of their children had gotten the vaccine. Geographically, 1 influencer lived in a rural area, 5 reported living suburban areas, and 4 reported residing in urban areas. 1 influencer began posting online between 3-5 years ago, 4 began posting between 5-10 years ago, and half of the influencers had been posting for more than 10 years. Seven of the 10 of influencers reported posting daily or almost daily, 2 reported posting once or twice a week, and 1 posted once or twice a month. Seven of the 10 influencers reported that Instagram was their preferred platform, 2 preferred TikTok, 1 preferred Twitter (now X), and 1 preferred to post on their blog. See Table 1.

**Table 1:** Demographic and sociographic characteristics of influencers.

		n (N = 10)	%
**Gender**	Female	10	100
	Male	0	0
**Race**	Caucasian	4	40
	African American	4	40
	No answer	2	20
**Ethnicity**	Non-Hispanic	2	20
	Hispanic	6	60
	No answer	2	20
**Marital Status**	Married	8	80
	Single, Engaged, Divorced, or Widowed	2	20
**Education Level**	HS Diploma	0	0
	Associate degree or some college	2	20
	College degree	7	70
	Graduate school/graduate degree	1	10
**Number of Children**	1 or 2	6	60
	3 or 4	3	30
	5 or more	1	10
**How hesitant about childhood shots would you consider yourself to be?**	Not at all hesitant	5	50
	Not too hesitant	3	30
	Somewhat hesitant	2	20
**Has your child/children gotten the HPV vaccine?**	Yes, at least one of my children has gotten the HPV vaccine	6	60
	No, none of my children have gotten the HPV vaccine	3	30
	I’m not sure if my child/ children have gotten the HPV vaccine	1	10
**Children’s Age** ^**^	Has 1 or more children between the ages of 0 and 8	5	50
	Has 1 or more children between the ages of 9 and 12	5	50
	Has 1 or more children 13 or older	8	80
**Geographic Residence**	Rural	1	10
	Suburban	5	50
	Urban	4	40
**Started Posting**	Less than 3 years ago	0	0
	Between 3 and 5 years ago (2017-2019)	1	10
	Between 5 and 10 years ago (2012-2018)	4	40
	More than 10 years ago	5	50
	Does not post	0	0
**Post Frequency**	Daily or almost daily	7	70
	Once or twice a week	2	20
	Once or twice a month	1	10
	Rarely/Does not post	0	0
**Preferred Platform**^	Instagram	7	70
	TikTok	2	20
	Twitter	1	10
	Blog	1	10

** The CDC recommends the HPV vaccine be given to children between the ages of 11 and 12. The vaccine can be given as early as 9 years old.

^ 1 participant selected more than one, so percentage will exceed 100.

#### EQ1: How did the influencers write about getting their children vaccinated against HPV in their posts?.

Influencers’ posts were highly emotional. Many began with an acknowledgement of fears that their followers may be facing, as one mom did, noting her worries about whether vaccinating her son against HPV would lead to him becoming sexually active at a young age because of the vaccine.

However, they all followed up this acknowledgement of fear with a discussion of how they made the decision to vaccinate. In many of the posts, influencers shared emotionally charged stories related to health issues or scares that helped motivate them. They also shared concerns from their community that prompted them to take the preventative step of vaccinating their children against HPV. One Latina influencer wrote,

*“Having experienced HPV myself, I fully understand the importance of the HPV vaccine.” – Participant 6*

This led to her decision to vaccinate her children, to:

*“… avoid the heartache and pain I went through, knowing it can help them have a healthy future.” – Participant 6*

Across all their posts, influencers shared their experiences as parents who want the best for their children, and shared facts around the safety and effectiveness of the HPV vaccine in its prevention of cervical cancer. One influencer wrote about how much “research [she conducted] and conviction [she exhibited]” in making this decision. Another noted how she came to the decision based on the evidence that the vaccine is effective, writing,

*“My decision is to get him vaccinated and protect him from any exposure to the HPV virus.” – Participant 2*

Another dimension of the emotional nature of this topic for these influencers was related to how the HPV vaccine seemed to mark a turning point in the growth and development of their child, and as a result, their responsibilities as a parent. Many expressed strong feelings of concern about whether they were making the right decision for their child. They emphasized wanting to make the best choice for their child – and how doing that is not always straightforward or easy. As one stated,

*“My child’s health is my responsibility until she can make her own informed decisions about her body, and I want to do the right thing.” – Participant 3*

Another noted,

*“Before having children, I never really thought about what being a mother entails. Of course, I thought about feeding them and clothing them. However, I’m talking about the harder things like should I vaccinate them or not. It honestly never occurred to me that this would be such a hard topic. You have so many people with different views on this topic and it can really get messy” – Participant 1*

They also noted how their children were growing up and how they were having to make big decisions that would impact them for the rest of their lives. These sentiments seemed tied to broader emotional feelings about their children growing up and having to navigate the world on their own, making important and life changing decisions. As one noted,

*“I can hardly believe the types of conversations we are having about my girls’ health. It seems like overnight we went from diapers to bigger issues like cervical cancer. I know the conversations will only get bigger from here, but it’s hard to face as a mom.” – Participant 3*

Another said,

*“We never want to think of the possibility of our children being exposed to a virus that can possibly alter their lives in the future” – Participant 2*

Related to this, it appeared that decision making around the HPV vaccine seemed to signal the big changes that were coming, and the lingering association with sexual activity made parents uncomfortable with the fact that the children were growing up and would become sexually active. As one noted in their interview,

*“… it’s one thing to think about, ‘Oh could my kid get sick from the ﬂu or from COVID?’ Yeah, probably, ‘cause they’re in spaces and it’s airborne and whatever, right? It’s another thing to think, ‘Could my kid get sick because of sexual activity,’ right? It’s like ahh nobody wants to think about that when they’re 9 and 12. [Laughs] So it’s that is what makes me feel like uncomfortable and just not want to think about it.” – Participant 7*

Finally, one noted in their post,

*“As a mom you can never really prepare yourself for these kinds of conversations with your children. The birds and the bees, the heartbreaks, that time of the month with your girls, but it is a must to help prepare them for the real world. Although I am vaccinated with the HPV vaccine, and I am pro vaccinations, I could not help thinking, maybe this is not a great idea for my children. My son is 14, and I want to keep him as innocent as possible. I don’t want him or my girls to experience the life that mom and dad did as teen parents. Yes, that was absolutely the thought that crossed my mind. Will my son become sexually active at a young age because of the vaccine?” – Participant 2*

Ultimately, the narrative storytelling approaches these influencers took with their posts helped convey important information about the topic of HPV vaccination, identified concerns that parents are having, and talked about solutions to help them feel like they are making the best decisions for their children.

#### EQ2: How clearly did the influencers recommend the HPV vaccine in their posts?.

As it relates to recommending the HPV vaccine, almost all of the influencers included in their posts something about their own process of decision making. They talked about how they sought out information about HPV and the vaccine and actively engaged with their providers and peers and searched online and in social media for information. In particular, they looked for storied and experiences from others.

In their posts, they then encouraged their audiences to do the same. They recommended that their followers do their own research and better educate themselves on the vaccine, highlighting facts around the disproportionate impact that HPV has on communities of color, especially how,

*“Hispanic women, as well as African American women, tend to have higher rates of HPV-related cancers than other women.” – Participant 8*

Few specifically included a direct call-to-action for their followers to get their children vaccinated. One noted,

*“Trust me, I know how hard it is to make these decisions. I highly recommend doing the research and knowing the facts. You’ll be happy you did.” – Participant 1*

Another noted,

*“This is called prevention, this is called self-care. And it’s important to urgently take action well before your children become sexually active. Do your own research, and consult with your doctor, and read upon on it because it’s super important. Prevention!” – Participant 10*

This is likely connected with the level of negative commentary and backlash that influencers can receive when they talk about vaccines online – especially for this vaccine, which is still seen as controversial despite it now being around for more than 20 years.

Several also stated that they did not want their audience to think that they were telling them what to do, so many emphasized that they framed their post to be their own personal opinion, not a didactic demand for others to vaccinate their children.

#### EQ3: How did the influencers perceive themselves in terms of their ability to be persuasive about vaccination to their followers?.

In terms of how the influencers perceived their ability to persuade their followers about the HPV vaccine, many noted the challenges sharing this kind of information online amidst the current debate about vaccination. They expressed how they would likely get negative commentary as a result of their posts. As one said,

*“Hispanic families… they feel uncomfortable and I always get hate no matter what. I always get one or two or three comments about that. They are always in Spanish. I mean I know probably I’m going to get hate.” – Participant 8*

Because of this, several were not sure that they would sway anyone who did not already hold those beliefs. As one noted,

*“You’re going to have some people that are just anti vaccines and they’re not going to budge.” – Participant 1*

Another said,

*“I mean, no. I think a lot of people don’t read. They don’t ask their doctors, and they just go by what they feel, and I respect that, but I think there’s a lot of misinformation. So, because I asked a little bit, I was like, “Do you guys have kids between those ages?” And most of the responses were, “I haven’t done it because I’m super scared.” Or many people have misconceptions. They’re like, “Oh, no. You’re giving permission to your kids to have sex.” I’m like, “I don’t see it at all that way,” but that’s a response I got. At least that’s what I felt that there was that.” – Participant 10*

Despite these concerns, most of the influencers in this study thought that their posts would influence followers who are “on the fence” about vaccinating their children to seek more information, talk to their doctors, or simply “strike some curiosity,” particularly those whose children were not yet of age. Others agreed that this topic was an important one to address, and that by sharing open and honest narratives with their followers, they could help pave the way towards positive change.

One said that she thought her post would help put the idea of the vaccine on the radar of her followers who have younger children and who might not yet be thinking about this vaccine,

*“I’m guessing that it will be a positive reaction… Like a lot of my followers have kids that are younger than nine so it wouldn’t really be on their radar yet.” – Participant 3*

Additionally, some thought that the posts would prompt their followers to learn more and do their own research,

*“I think it will strike curiosity about the HPV vaccine for those who are hearing about it. So yeah, I think it would definitely strike some curiosity and have them think about HPV in general, and how if there’s something to prevent this, let’s go see what that is.” – Participant 9*

Still another,

*“I hope. I mean I don’t wanna change their mind. I want them to go and ask their doctors and read. I wanna get into their heads to say ‘This is what’s happening. You wanna do the research? What do you wanna see?’ I want them to be like, ‘What is she talking about? I’m going to ask my doctor. Is this for real? What is she saying?’ and then that’s why I keep saying, ‘Talk to your doctor. Talk to your doctor.’” – Participant 10*

Moreover, several did, indeed, think that their posts could and would change people’s minds. As one noted,

*“I think I will, especially if it’s someone who is hesitant about getting it. But I also feel like it will be some people who weren’t aware of it at all. Believe or not I do feel like there’s a lot of people who do not know about it at all.” – Participant 2*

Another noted,

*“I think there’s definitely people that are hesitant and I would hope that sharing my story would just help them to do more research, so that might change their mind a little bit, yes.” – Participant 3*

Another noted,

*“The people that are on the fence, I do think that it would be something that they would at least consider now, because I feel like when you’re on the fence that means that you’re still able to persuade one way or the other. And the fact that I know that I have persuaded on things before – not necessarily the vaccine – you know, I inﬂuence them just with anything that I do. And I have those DMs and messages to prove that, ‘Hey, I went and picked up this. Oh, I never even thought about this.’” – Participant 1*

And another,

*“I think they’ll react, for the most part, pretty positively. I think it will change some minds. I really hope it will. I don’t know how vocal people will be about it changing their minds. With things like this – things – topics that are a little more, let’s say, taboo on Instagram, people will tend to do things like they’ll message me later on and be like, hey, thanks for that that you posted. You know, when we went to the doctor, we talked to him about it. But, it’ll be down the line – not immediately. But I think it will, and I think it’ll help. I think it’ll at least help a lot of people want to look into it, which is what I’m hoping will happen I do think that there are people who are on the fence that may need this post to kind of push them over to getting their kids vaccinated.” – Participant 5*

Even those who thought they would get angry responses or hate messages, still thought that it was worth talking about, even with all the negativity:

*“But it’s important to face this topic and it’s okay. I mean the more we talk more people can get you know educated information, where and how… Yeah as soon as I saw it I say, “You know what I can do this and it’s fine and I can, I can talk about my personal experience.” – Participant 8*

Ultimately, once the posts went live, many reported that their followers did, in fact, react positively to their posts and even in some cases, sent Direct Messages (DMs) responding positively to the information that was shared.

## Discussion

This study contributes to the field of health communication by providing new data and supporting evidence related to the powerful roles of opinion leaders and narrative messaging in HPV vaccine communication. Specifically, findings from this study suggest that the interaction of persuasive opinion leaders and personal or narrative storytelling can enable the successful sharing of supportive messaging about HPV vaccination with parents and communities of color.

Opinion leaders were first defined by Rogers [46] in his Diffusion of Innovations theory, and since then, there has been a robust literature supporting the successful use of opinion leaders in public health prevention programs to influence behavior change [47]. As media channels evolved, the advent of the Internet and social media have enabled the growth a new type of opinion leader – that is, social media influencers [8]. Culture has always played a key role in the relationship between opinion leaders and their communities. However, as we talk about health communication and equitable and inclusive access to health care, culture plays an increasingly important role in supporting positive health outcomes, especially for innovations such as the HPV vaccine [48–50].

In today’s context, intermediaries need to be identified intentionally, paying close attention to the cultural and/or linguistic “fit” with their followers [51]. This, in turn, can help ensure that messages are developed with the relevance and context needed to resonate with their communities.

As well, Narrative Theory is based on the premise that narrative messaging, or storytelling, is a basic way that humans process our lived experiences [52]; and the rich body of work around narrative storytelling documents its positive effects on health behavior change, especially when paired with didactic methods [53]. It examines the various structures, elements, uses, and effects of narrative storytelling.

According to Narrative Theory, personal narratives create an openness to accept new information, bypassing the theory that doubt can often be attributed to a lack of knowledge [54]. Moreover, this theory has been documented as a communication approach that can effectively identify narratives around HPV vaccination, contributing to culturally relevant interventions, which, in turn, led to higher HPV vaccination rates [50].

This study’s findings that social media influencers positively messaged about the HPV vaccine using personal storytelling and considered themselves to be persuasive, despite the sensitive and emotional nature of the topic, support these points in several ways.

First, the study’s approach to recruitment was intentional. The study team partnered with a women- and minority-owned company specifically to recruit Hispanic, Black, Asian, and Native American influencers, and ensure that the posts would reach these communities. Given the disproportionately higher rates of HPV-related diseases and the lower rates of HPV vaccination amongst these communities, identifying messengers who are from these communities, who can write about this topic in ways that are meaningful to them, and therefore, be persuasive to them, is key.

As well, the influencers’ approaches to HPV vaccination messaging included the use of storytelling to convey HPV vaccine information. They shared the evidence-based messages within the context of their own experiences as parents who were faced with the same important health decision on behalf of their children and/or individuals who had faced related health crises and how the vaccine is a powerful prevention tool.

Moreover, we found that the influencers expressed very similar views on the vaccine in both their interviews and posts; however, in their interviews, some discussed further how tailoring their messaging using their own personal experiences can help make the health message more digestible for their followers. This was evinced in several of the posts, where influencers gently introduced this sensitive topic of HPV vaccination by sharing their individual stories of experiences with HPV-related diseases and the vaccine, and ultimately, their decisions to vaccinate.

These findings align with what we know about opinion leadership and health communication – that they are experts in reaching their communities effectively, and thus know best how to share evidence-based health information in ways that will resonate with them. They also align with what we know about narrative storytelling, that is, for this kind of messaging to be effective, the messengers must have the freedom to speak and write from their own perspective – and this is especially important when it comes to social media influencers [8].

This study intentionally embraced this approach – providing the influencers with evidence-based information (in the form of the fact sheet) while still allowing for influencers to tailor and personalize their messages. This approach not only is documented to be effective, but it also ensured that the key medical information was shared accurately while still allowing for full creative control of the story by the influencer.

Ultimately, this study demonstrates how partnering with influencers of color who are trusted by their followers and who are willing to share personal and tailored narratives about the HPV vaccine enables the sharing of evidence-based messages. It also showcases how these influencers see themselves as persuasive and how, in fact, their followers are open to and react positively to this kind of information, when framed in this way. Thus, intentionally partnering with online influencers of color to share evidence-based HPV vaccine information using personal storytelling approaches is a possible way to successfully communicate about this topic at scale.

### Limitations

This study is not without limitations. First, findings were extrapolated from a sample of 10 influencers, the majority of which were married (8 out of 10) and received a higher education (9 out of 10). While this sample is still adequate for rich description of the study’s aims, future studies should consider diversifying the sample of participating influencers and could also increasing the sample size of influencers engaged, where possible Second, qualitatively, the study did not explore the effects of the influencers’ messages on their followers, therefore, the perspectives presented in this paper are only those of the influencers. Finally, it is important to note the potential role of bias in this study. As noted, the sample could have introduced certain biases into these findings due to its composition. A different sample might reveal unique perspectives not reflected here. As well, as a qualitative study, the interpretation of the data may be influenced by certain biases within the study team. The team attempted to avoid such risk of bias by triangulating findings across the team and assessing both interview- and post-related data. That said, additional analyses and incorporation of other data sets (e.g., quantitative, secondary data) would help address this risk in future studies.

## Conclusions

Despite its effectiveness in preventing cervical cancer, the HPV vaccine has been a difficult topic to navigate, particularly among communities of color. Leveraging trusted voices through the integration of social media influencers has become a well-documented practice in public health communication literature. Given the disproportionate impacts of cervical cancer on communities of color, this study aimed to explore how influencers of color can be effective, trusted messengers of HPV vaccination messages. The study findings suggest that narrative storytelling provided via trust messengers such as social media influencers can be a powerful way for parents to connect around the emotional nuance and uncertainty of the HPV vaccine and its implications. These findings may help inform current and future programs aiming to increase the rates of HPV vaccination among these communities.

## Supporting information

S1 Appendix Influencer recruitment email. (DOCX)

S2 Appendix Screening questions for influencer recruitment. (DOCX)

S3 Appendix Verbal consent for telephone interview. (DOCX)

S4 Appendix Influencer interview guide. (DOCX)

S5 Appendix HPV vaccine research study – Vaccine hesitancy project guidelines. (DOCX)

S6 Appendix Facts at your fingertips – HPV information for influencers. (DOCX)
