# Supplementary material for: Findings from a qualitative analysis: Social media influencers of color as trusted messengers of HPV vaccination messages
Source: PLoS One. 2025 Apr 4;20(4):e0319160. doi: 10.1371/journal.pone.0319160 (PMC11970659; doi:10.1371/journal.pone.0319160)
Supplement: S1 Appendix — (DOCX) [file pone.0319160.s001.docx]

**S1 Appendix. Influencer Recruitment Email**

Initial Email:

*Dear [insert name of influencer]:*

*I hope that my email finds you well! [insert any tailoring based on personal history with this influencer]*

*I am reaching out as my company Ruiz Family Enterprises, LLC is working with Thomas Jefferson University and the NORC at the University of Chicago to recruit influencers like yourself for a timely research study around HPV (Human papillomavirus). The study is funded by Merck and seeks to better understand HPV vaccine hesitancy among communities of color, which have been disproportionately affected by the pandemic.*

***For this research project, we are looking for Hispanic, Black, Asian, or Native American parents or caregivers of children between the ages of 9-14.***

*We know that you play an important role in decisions that are made about the health of you and your family, and that your opinions matter to your network of followers. As such, we would like to invite you to be part of this study to help us (1) to understand how to create pro-vaccine messages that reach and resonate with communities of color and (2) to assess how social media messages and messengers impact trust and vaccine acceptance in these communities.*

*Participation in this study will include the following:*

1. *Develop* ***one (1) social or blog post*** *encouraging vaccination in your own words and submit for review by the research team. Detailed guidelines will be provided if you agree to participate.*
2. *Participate in* ***one (1) 30-minute*** *phone interview regarding your blog post. Anything that you say will be kept confidential.*
3. ***Identify and recruit 20 of your followers*** *to participate in* ***one (1) short online survey*** *for which they will be compensated $55. Recruitment messages that you will share with your followers will be provided by our team. After all 20 followers complete the survey, your blog post will go live and our team will gather additional engagement data from the public.*
4. *All activities will take place between* [*insert project dates from when first call will take place to when follower surveys will be completed]. Please note that these are estimated dates and may shift.*

*To compensate you for your time, we are offering a payment of [insert value depending on influencer]. We can also share with you all results from the work we do once they are available. Due to the sensitivity of this study, we ask that you please keep all information regarding this project private until we are able to share the results more widely.*

*We greatly appreciate you considering this opportunity. Your participation would help us grow our understanding of how to promote COVID-19 vaccines to communities of color, a segment of the U.S. population that has disproportionately borne the brunt of this historic pandemic.*

*Please reach out with any questions or if you would like to discuss further via phone.*

*Thank you,*

*[insert name of influencer recruiter]*

Follow-Up Email if no Response:

*Dear [insert name of influencer]:*

*I wanted to take a moment to quickly follow up on my previous email. When you have a moment, please let me know if you are interested in finding out more and possibly participating.*

*Thank you,*

*[insert name of influencer recruiter]*

Follow-Up Email if no Interest from Influencer:

*Hi [insert name of influencer]:*

*Thank you so much for getting back to us. We understand that you do not wish to be involved. Please feel free to let us know if anything changes.*

*Thank you,*

*[insert name of influencer recruiter]*

Email for Influencer to Publish Post:

*Hi [insert name of influencer]:*

*Thank you for submitting your post for the research team to review. The review process has been completed, and we’ve confirmed that 20 of your followers have completed the online survey. You can now publish and promote your post.*

*Please reach out with any questions.*

*Thank you,*

*[insert name of influencer recruiter]*

Thank You Email for Influencer:

*Hi [insert name of influencer]:*

*Thank you for participating in this important and timely research study around HPV. Your participation will help us to better understand how to promote HPV vaccines to communities of color.*

*Please remember that due to the sensitivity of this study, we ask that you keep all information regarding this project private until we are able to share the results more widely. We are happy to share final study results with you once they are available, at which point you will be welcome to circulate and share with your community.*

*Thank you,*

*[insert name of influencer recruiter]*
