# Supplementary material for: Findings from a qualitative analysis: Social media influencers of color as trusted messengers of HPV vaccination messages
Source: PLoS One. 2025 Apr 4;20(4):e0319160. doi: 10.1371/journal.pone.0319160 (PMC11970659; doi:10.1371/journal.pone.0319160)
Supplement: S2 Appendix — (DOCX) [file pone.0319160.s002.docx]

**S2 Appendix. Screening Questions for Influencer Recruitment**

**Eligibility for Merck Influencers (Questions 1-4):**

- Having a child/children ages 9-14
- Being from a minority race/ethnic background
- Having positive attitudes about vaccines for children
- Willing to write about the HPV vaccine on their social media platform

1. Do you have a child or children between the ages of 9 and 14?
2. Yes
3. No -> STOP, not eligible.

2. Which of the following races/ethnicities describes your household (choose all that apply):

- 1. Hispanic or Latino
  2. African American
  3. Native American or American Indian
  4. Asian/Pacific Islander
  5. Caucasian -> STOP, not eligible if ONLY Caucasian
  6. Other: ______________________________
  7. Prefer not to answer -> STOP, not eligible

3. Overall, how hesitant about childhood shots would you consider yourself to be?

a. Not at all hesitant

b. Not too hesitant

c. Not sure

d. Somewhat hesitant -> STOP, not eligible

e. Very hesitant -> STOP, not eligible

4. Are you willing to write about the HPV vaccine on your social media, blog, or other digital platform?

1. Yes
2. No -> STOP, not eligible

FOR THOSE WHO PASS THE SCREENER QUESTIONS:

5. Has your child or children gotten the HPV vaccine?

a. Yes, at least one of my children has gotten the HPV vaccine

b. No, none of my children have gotten the HPV vaccine

c. I’m not sure if my child / children have gotten the HPV vaccine

6. What is your gender?

a. Male

b. Female

c. Non-binary

d. Transgender

e. Other

7. What is the highest degree or level of school you have completed?

a. Less than a high school diploma

b. A high school diploma/ GED

c. An associate degree, trade school, or some college

d. A college degree

e. A post-college or graduate degree

8. What is your household income?

a. Less than $20,000

b. $20,000 - $40,000

c. $40,000 - $60,000

c. $60,000 - $80,000

d. $80,000 - $100,00

e. Greater than $100,000

f. Prefer not to answer

9. Please fill out the following contact information:

1. Name:
2. Blog or platform (e.g., podcast, website) name:
3. Primary social media handle (URL):
4. Email address:
5. City:
6. State:
