# Supplementary material for: Findings from a qualitative analysis: Social media influencers of color as trusted messengers of HPV vaccination messages
Source: PLoS One. 2025 Apr 4;20(4):e0319160. doi: 10.1371/journal.pone.0319160 (PMC11970659; doi:10.1371/journal.pone.0319160)
Supplement: S3 Appendix — (DOCX) [file pone.0319160.s003.docx]

**S3 Appendix. Verbal Consent for Telephone Interview**

Thank you for taking the time to speak with me today.

The purpose of this interview is to understand your thoughts about how you wrote your HPV vaccine post.

I am the interviewer and you are the expert – all of your thoughts and responses are appreciated. I will remain neutral to the topic throughout the interview.

I would like to record this conversation. Is that ok?

*Yes (Agree)*

*No(Disagree)*

[If Yes] *Begin recording.*

**Before we get started, I am going to read you a brief consent statement to ensure that you want to participate in this research. OK?**

Thank you for agreeing to be interviewed. We are eager to hear your thoughts about how you wrote your HPV vaccine post. Your participation is entirely voluntary. You may stop the interview at any time or decline to answer a specific question.

The risks of partaking in this study are very low. If you become uncomfortable or anxious as a result of any of the questions, please let us know and we will immediately pause the interview. In addition to this risk, there may also be risks that are not known at this time.

You may not personally benefit from taking part in this research, but other people may be helped by what is learned.

We want to assure you that any information you provide today will remain strictly confidential. Your name will not be identified or associated with any specific responses, and it will not appear in any published materials which result from this research.

If you have questions about this research, you can call Dr. Amy Leader at 215-955-7739 or email her at [amy.leader@jefferson.edu](mailto:amy.leader@jefferson.edu). Also, if you have questions about your rights as a research participant, you can contact the Thomas Jefferson University Office of Human Research at 215-503-8966.

**Would you like to participate in the study?**

*Yes (Agree)*

*No(Disagree)*

Ok, let’s get started.
