# Supplementary material for: Findings from a qualitative analysis: Social media influencers of color as trusted messengers of HPV vaccination messages
Source: PLoS One. 2025 Apr 4;20(4):e0319160. doi: 10.1371/journal.pone.0319160 (PMC11970659; doi:10.1371/journal.pone.0319160)
Supplement: S4 Appendix — (DOCX) [file pone.0319160.s004.docx]

**S4 Appendix. Influencer Interview Guide**

**Introduction:**

Thank you for taking the time to speak with me today. The purpose of this interview is to understand the thought process behind your blog post about HPV vaccination. I am the interviewer and you are the expert – all of your thoughts and responses are appreciated. I will remain neutral to the topic throughout the interview. Let’s get started.

**Background:**

Let’s start at the beginning. Can you tell me a little bit about yourself and your family? How many children do you have? What are their genders?

**Discussion about Blogging:**

When did you start posting on social media? What was your original purpose and has it changed over time?

Who is your typical audience?

Do you ever post about health topics? If so, which ones?

What sources do you feel are most trustworthy for parenting information, particularly when making health decisions? [We may want to probe specifically: health care professionals (in general), their child’s health care professionals, other parents, government sources, certain blogs/websites/social media accounts, etc.).]

Do you look to other people’s posts for parenting information and advice? Do you think people look to your posts for parenting information or advice?

Prior to this post about the HPV vaccine, have you written about vaccines on your social media platforms? If so, which vaccines? What was your message? What was the reaction from your followers?

**Discussion about HPV and Vaccination:**

In general, what are your views on the HPV vaccine? Do you think your audience shares those same views?

How has the pandemic effected your views on the HPV vaccine? (Have you thought about it? /Is it a priority right now?)

In your physical community (not on social media), do you think the people closest to you share those same views?

Prior to this post, had you written about the HPV vaccine on your social media platforms? If so, what had you written about?

Did you have any concerns about taking on this assignment to write about the HPV vaccine on your blog post? If yes, what ultimately made you decide to take the assignment?

**Creating the Post**

When you first got this assignment, what were your initial thoughts or ideas for the post?

What did you think was going to be the most challenging part about writing this post?

Can you describe your post to me? Can you talk me through your thought process in creating it?

Did you use any of the resources or information that we provided to you? Why or why not?


Did you do any research for your post, beyond what we shared with you? If yes, where did you go to find that information?

Were there any misconceptions or thoughts that you were specifically aiming to address or overcome with your post?

Were there specific things (words, images, etc.) that you knew you wanted to include? What were those, and why?

Did you include any personal stories in your post? If so, what was it about and why did you choose it?

What platforms do you plan to post this on? Why those platforms? Why not others?

How do you think your followers will react to your post? Do you think it will change any minds about HPV vaccine? Why or why not?

Thank you for your time. I only have a few questions about you:

1. Age _______

2. Gender/age of children ____________________________________________

3. What state do you live in?____________

4. Would you say that you live in a rural, urban or suburban area?________________

5. What is your race and ethnicity?_____________________

6. Your highest level of education? _________________________

7. Are you single, married, or divorced? _____________________________

8. When did you start posting on social media? ____________________________

9. What is your preferred social media platform? __________________________

10. Number of followers ________________________

11. How often do you post?_____________________________
