# Supplementary material for: Findings from a qualitative analysis: Social media influencers of color as trusted messengers of HPV vaccination messages
Source: PLoS One. 2025 Apr 4;20(4):e0319160. doi: 10.1371/journal.pone.0319160 (PMC11970659; doi:10.1371/journal.pone.0319160)
Supplement: S5 Appendix — (DOCX) [file pone.0319160.s005.docx]

**S5 Appendix. HPV Vaccine Research Study – Vaccine Hesitancy Project Guidelines**

| **Project** | **HPV Research Study Vaccine Hesitancy Among Communities of Color** |
| --- | --- |
| **Research Project Participant Criteria** | **Who is eligible to participate in this research study?**  For this research project, we are looking for Hispanic, Black, Asian, or Native American parents or caregivers of children between the ages of 9-14. |
| **Research Study Overview** | **What is this HPV research study about?**  Thomas Jefferson University and NORC at the University of Chicago are working with a select group of influencers for a timely research study around HPV. NORC at the University of Chicago is an objective, nonpartisan, research organization that deliver​s reliable data and rigorous analysis.  The study is funded by Merck and seeks to better understand HPV vaccine hesitancy rates among communities of color, which have been disproportionately affected by the pandemic. Specifically, the study focuses on parents and caregivers getting their adolescent children ages 9-14 vaccinated. The one-year study will focus on analyzing your content and surveying your followers on their perceptions before and after they read your blog post.  Your participation in this study will help us (1) to understand how to create pro-vaccine messages that reach and resonate with communities of color and (2) to assess how social media messages and messengers impact trust and vaccine acceptance in these communities.  **Due to the sensitivity of this study, we ask that you please keep all information regarding this project private until we are able to share the results more widely.** |
| **What to Avoid** | Please note the following when planning out your content:   - Do not include other people in your photo/video content - Do not wear any visible brand logos, names etc. - Do not include copyrighted material or music - No references to excessive drinking, partying, or hangovers - No profanity or nudity - No references to behavior that may be risky during the COVID-19 pandemic (i.e. gathering in groups, going to large parties, going out to restaurants, traveling |
| **Compensation** | Compensation for influencers will be coordinated as outlined in your project agreement.  Compensation for participating followers will be made by Ruiz Family Enterprises, LLC upon completion of the short online survey.  For any questions regarding compensation, please contact Angela Sustaita-Ruiz at [angela.sustaitaruiz@gmail.com](mailto:angela.sustaitaruiz@gmail.com). |
