# Supplementary material for: Findings from a qualitative analysis: Social media influencers of color as trusted messengers of HPV vaccination messages
Source: PLoS One. 2025 Apr 4;20(4):e0319160. doi: 10.1371/journal.pone.0319160 (PMC11970659; doi:10.1371/journal.pone.0319160)
Supplement: S6 Appendix — (DOCX) [file pone.0319160.s006.docx]

**S6 Appendix. Facts at your Fingertips – HPV Information for Influencers**

| **HPV VACCINE FACTS AT YOUR FINGERTIPS**  ***NOTE:*** *This information may be helpful when creating your post. You are not required to use any of this information; similarly, do not copy the entirety of this information directly into your post.* | |
| --- | --- |
| **Overview** | - Human Papillomavirus (HPV) is a virus that may cause genital warts and certain cancers. - HPV causes 90% of cervical cancer in women and 65% of penile cancer in men. - These infections are often transmitted sexually or through other skin-to-skin contact. - There is currently no cure for HPV. |
| **What is the State of HPV Infections?** | - About 45,300 HPV-associated cancers occur in the United States (US) each year. - 4 out of 5 people will have had an HPV infection at some point. - Black and Hispanic women have higher rates of HPV-related cancers than other women - Hispanic men have higher rates of HPV-related penile cancer than men of other races or ethnicities |
| **What is Known about the HPV Vaccine?** | - The HPV vaccine is approved by the U.S. Food and Drug Administration for both girls and boys. - The vaccine protects against 9 different types of HPV that cause cancer in men and women. - The Centers for Disease Control and Prevention (CDC) recommends that the HPV vaccine is given to girls and boys between the ages of 11 and 12. - The vaccine can be given as early as 9 years old. - It is safe for the COVID-19 vaccine to be given at the same time as the HPV vaccine |
| **Why Should I Vaccinate My Child against HPV?** | - The vaccine is 95% effective in preventing cervical cancer in women. - The vaccine is 90% effective in preventing genital warts and 75% effective in preventing anal pre-cancers in men. - It is ideal for girls and boys to receive the vaccine before they have sexual contact and are exposed to HPV. Research has shown that receiving the vaccine at a young age isn't linked to an earlier start of sexual activity. |
| **What are the Risks of Vaccinating My Child?** | - The HPV vaccine has been found to be safe in many studies. - Overall, the side effects are usually mild. The most common side effects of the HPV vaccine is soreness, swelling or redness at the injection site. - Sometimes dizziness or fainting occurs after the injection. Remaining seated for 15 minutes after the injection can reduce the risk of fainting. Headaches, nausea, vomiting, fatigue or weakness also may occur. |
| **More Information and Sources** | **American Academy of Pediatrics (AAP):**  <https://www.aap.org/en/pages/2019-novel-coronavirus-covid-19-infections/children-and-covid-19-state-level-data-report/>  <https://www.aafp.org/dam/AAFP/documents/patient_care/immunizations/hpv-recommendation-letter.pdf>  **Centers for Disease Control and Prevention (CDC):**  <https://www.cdc.gov/cancer/hpv/statistics/race.htm>  **Mayo Clinic:**  <https://www.mayoclinic.org/diseases-conditions/hpv-infection/symptoms-causes/syc-20351596>  <https://www.mayoclinic.org/diseases-conditions/hpv-infection/in-depth/hpv-vaccine/art-20047292>  <https://www.mayoclinic.org/diseases-conditions/hpv-infection/in-depth/hpv-vaccine/art-20047292#:~:text=Gardasil%209%20is%20an%20HPV,prevent%20vaginal%20and%20vulvar%20cancer>  **MD Anderson:**  <https://www.mdanderson.org/publications/focused-on-health/i-have-hpv--now-what-.h13-1592991.html> |
